# In vivo protein expression changes in mouse livers treated with dialyzed coffee extract as determined by IP-HPLC

**DOI:** 10.1186/s40902-018-0183-z

**Published:** 2018-12-28

**Authors:** Cheol Soo Yoon, Min Keun Kim, Yeon Sook Kim, Suk Keun Lee

**Affiliations:** 1 Department of Oral Pathology, College of Dentistry, Gangneung-Wonju National University and Institute of Oral Science, 123 Chibyun-dong, Gangneung, 210-702 South Korea; 2 Department of Oral and Maxillofacial Surgery, College of Dentistry, Gangneung-Wonju National University and Institute of Oral Science, Gangneung, South Korea; 3 Department of Dental Hygiene, College of Health Sciences, Cheongju University, Cheongju, South Korea

**Keywords:** Murine hepatocytes, Protein expressions, Cellular regeneration, IP-HPLC

## Abstract

**Background:**

Coffee extract has been investigated by many authors, and many minor components of coffee are known, such as polyphenols, diterpenes (kahweol and cafestol), melanoidins, and trigonelline, to have anti-inflammatory, anti-oxidant, anti-angiogenic, anticancer, chemoprotective, and hepatoprotective effects. Therefore, it is necessary to know its pharmacological effect on hepatocytes which show the most active cellular regeneration in body.

**Methods:**

In order to determine whether coffee extract has a beneficial effect on the liver, 20 C57BL/6J mice were intraperitoneally injected once with dialyzed coffee extract (DCE)-2.5 (equivalent to 2.5 cups of coffee a day in man), DCE-5, or DCE-10, or normal saline (control), and then followed by histological observation and IP-HPLC (immunoprecipitation high performance liquid chromatography) over 24 h.

**Results:**

Mice treated with DCE-2.5 or DCE-5 showed markedly hypertrophic hepatocytes with eosinophilic cytoplasms, while those treated with DCE-10 showed slightly hypertrophic hepatocytes, which were well aligned in hepatic cords with increased sinusoidal spaces. DCE induced the upregulations of cellular proliferation, growth factor/RAS signaling, cellular protection, p53-mediated apoptosis, angiogenesis, and antioxidant and protection-related proteins, and the downregulations of NFkB signaling proteins, inflammatory proteins, and oncogenic proteins in mouse livers. These protein expression changes induced by DCE were usually limited to the range ± 10%, suggesting murine hepatocytes were safely reactive to DCE within the threshold of physiological homeostasis. DCE-2.5 and DCE-5 induced relatively mild dose-dependent changes in protein expressions for cellular regeneration and de novo angiogenesis as compared with non-treated controls, whereas DCE-10 induced fluctuations in protein expressions.

**Conclusion:**

These observations suggested that DCE-2.5 and DCE-5 were safer and more beneficial to murine hepatocytes than DCE-10. It was also found that murine hepatocytes treated with DCE showed mild p53-mediated apoptosis, followed by cellular proliferation and growth devoid of fibrosis signaling (as determined by IP-HPLC), and subsequently progressed to rapid cellular regeneration and wound healing in the absence of any inflammatory reaction based on histologic observations.

**Electronic supplementary material:**

The online version of this article (10.1186/s40902-018-0183-z) contains supplementary material, which is available to authorized users.

## Background

Coffee is one of the most popular daily drinks not only for ordinary people but also for patients in convalescence period after major surgical treatment, but too much coffee may harm human health. However, published results are controversial with respect to its effects on cardiovascular diseases, inflammation, diabetes, Parkinson’s disease, cancer, and other diseases [1–3]. The beneficial pharmacological effects of coffee mentioned in the literature include anti-inflammatory, anti-oxidant, anti-angiogenic, anticancer, chemoprotective, and hepatoprotective effects [4–7], and coffee has been reported to contain polyphenols, diterpenes (kahweol and cafestol), melanoidins, and trigonelline [8–11].

Epidemiological studies support associations between coffee-specific diterpenes and various beneficial health effects. Although free cafestol and kahweol (coffee-specific diterpenes) have been recently reported to have antiangiogenic properties, little data is available regarding the health effects of esterified cafestol and kahweol, in particular, of their palmitate esters, which are the main diterpene esters present in coffee. Cafestol and kahweol palmitates inhibit the angiogenesis of human microvascular endothelial cells (HMVECs), although the effect of the kahweol ester is greater [12]. Kahweol has also been reported to protect against liver inflammation by downregulating the expressions of LPS-stimulated phospho-nuclear factor kappa B (NFkB) and signal transducer and activator of transcription 3 (STAT-3) expression [13].

Polyphenols derived from coffee beans have beneficial effects on blood pressure and vascular endothelial function, improve skin condition, and participate in cutaneous blood flow regulation after cold stress [14]. Chlorogenic acid hydrogels applied topically to significantly reduced wound areas during the inflammatory phase, possibly because of the well-known antioxidant and anti-inflammatory effects of chlorogenic acid, whereas caffeine (a known anti-oxidant) impeded keratinocyte proliferation and migration, suggesting it had an inhibitory effect on wound healing and epithelialization [15].

In our previous study, dialyzed coffee extract (DCE) and artificial coffee (AC) induced protein expressions in RAW 264.7 cells were compared by IP-HPLC analysis. DCE, which contains most of the minor components of coffee (including chlorogenic acid and caffeine), induced the expressions of proteins required for essential cellular functions in RAW 264.7 cells, while AC (1 mM chlorogenic acid and 2 mM caffeine, which are the same concentrations found in DCE) induced a quite different protein expressional pattern as determined by IP-HPLC. DCE caused the upregulations of proteins associated with cellular proliferation and protection, and antioxidant-related proteins, and the downregulations of apoptosis-related, angiogenesis-related, and oncogenic proteins; and enhanced cMyc/MAX, Rb/E2F, and RAS, growth factor signaling as well as osteogenesis in RAW 264.7 cells. Actually, overall protein expressional changes after DCE treatment revealed a signaling circuit triggered by antioxidant-related proteins and genetic/epigenetic activation [16].

The present study was undertaken to examine changes in protein expressions in mouse liver after administering animals the equivalents of 2.5, 5, or 10 cups of coffee in man (DCE-2.5, DCE-5, or DCE-10, respectively; determined on a body weight basis) to determine whether coffee has a beneficial effect on the liver. Mice were injected intraperitoneally with DCE to avoid issues associated with gastrointestinal absorption variability, and global protein expressions in mouse livers obtained 24 h later were determined by IP-HPLC.

## Methods

### Production of dialyzed coffee extract (DCE)

Twenty cups of coffee (20 × 150 mL = 3000 mL) were prepared from medium roasted coffee beans (*Coffea arabica* L., Nepal, at 20 g per cup and 90–95 °C. Aliquots (300 mL) of this extract were repeatedly dialyzed 10 times using a permeable cellulose bag (< 1000 Da; 131,492, Spectra, USA) in 1500 mL of double distilled water at 4 °C with stirring for 2 h. The dialyzed coffee extract (DCE) was immediately stored at − 70 °C until use.

DCE was subjected to non-adherent reverse phase column chromatography (YMC-Pak, Japan) using water as an eluent and a HPLC unit (1100, Agilent, USA). It was found that the primary constituents of DCE were caffeine and chlorogenic acid. HPLC analysis of DCE revealed a caffeine concentration of ~ 2 mM, indicating that 150 mL DCE contained ~ 60 mg of caffeine (Table 1). Because 150 mL of ordinary coffee extract contained ~ 120 mg of caffeine, the dialysis coefficient for the caffeine of coffee was ~ 50% and 300 mL of DCE was equivalent to one cup of coffee extract (150 mL) for a human adult (mean 60 kg, 59.4 l). Thus, 300 mL of DCE for a human adult (DCE-1) was equivalent to 0.15 mL of DCE for a mouse (mean 30 g) in animal experiment (Table 1).

**Table 1:** Calculation of DCE doses for a mouse to achieve equivalence with human adults

Coffee	Ordinary coffee extract	Dialyzed coffee extract (DCE)
Caffeine concentration	120 mg/150 mL	60 mg/150 mL
Dialysis coefficient		50%
One cup for a human adult (60 kg, 59.4 L)	150 mL	300 mL (DCE-1)
DCE-1 to 30 g mouse		0.15 mL
DCE-2.5 to 30 g mouse		0.375 mL
DCE-5 to 30 g mouse		0.75 mL
DCE-10 to 30 g mouse		1.5 mL

In order to check for lipopolysaccharide (LPS) contamination in DCE, a LPS detection assay was performed by IP-HPLC using anti-LPS antibody (Santa Cruz Biotechnology, USA). DCE (1 and 2 mL, experiments 1 and 2), 1 mL LPS solution (1 ng/mL, Sigma Aldrich, USA, positive control), and 1 mL distilled water (negative control) were separately analyzed by IP-HPLC. Peak areas of experiments 1 and 2 were similar to that of the negative control, while the peak area of the positive control (LPS solution) was predominantly increased (Additional file 1) [16]. These results indicated DCE was effectively free from LPS contamination.

### Mouse experiment

Twenty 9-week-old male, specific pathogen-free C57BL/6J mice were allocated to four groups, that is, to DCE-2.5 (*n* = 5), DCE-5 (*n* = 5), or DCE-10 (*n* = 5) groups or a control group (*n* = 5). Each mouse was intraperitoneally (i.p.) injected with DCE-2.5 (0.375 mL DCE, equivalent to 2.5 cups of coffee for an adult), DCE-5 (0.75 mL DCE), or DCE-10 (1.5 mL DCE), or 1 mL of normal saline. Mice were euthanized 24 h later, and their livers were removed for protein and histological analysis.

A sample of liver, measuring ~10 × 10 × 10 mm, was homogenized and lysed with protein lysis buffer (PRO-PREP™, iNtRON Biotechnology, INC, South Korea) and was immediately preserved at − 70 °C until required. In addition, another liver sample, measuring ~15 × 15 × 15 mm, was fixed in 10% neutral formalin, paraffin wax embedded, and sectioned at 4 μm.

### Histological and immunohistochemical observation

Mouse liver microsections in 4 μm thickness were subjected to hematoxylin and eosin staining, and serial microsections were also prepared for immunohistochemical staining using representative antisera of hepatocyte growth factor (HGF), glutathione S-transferase-1 (GST-1), poly-ADP ribose polymerase (PARP), or tumor necrosis factor-α (TNFα) (Santa Cruz Biotech. USA). Immunohistochemical reaction protocols differed according to target antigens and manufacturers’ protocols, but briefly, after xylene deparaffinization and rehydration in an ethanol series, sections were incubated with 0.5% hydrogen peroxide in phosphate-buffered saline for 30 min. Primary anti-human (rabbit/mouse/goat) polyclonal antibodies were applied to each section using triple sandwich indirect immunohistochemical methods [17]. Histochemical stains were observed under a light microscope, and images were captured by a digital camera (DP-73; Olympus Co., Japan).

### Immunoprecipitation high performance liquid chromatography (IP-HPLC)

Protein extracts (~ 100 μg) were immunoprecipitated using protein A/G agarose columns (Amicogen, South Korea), which were separately pre-incubated with 1 μg of 197 different antisera, including proliferation-related proteins (*n* = 11), cMyc/MAX/MAD network proteins (*n* = 3), p53/Rb/E2F signaling proteins (*n* = 5 (1)), epigenetic modification proteins (*n* = 6), protein translation proteins (*n* = 5), growth factors (*n* = 16), RAS signaling proteins (*n* = 12), NFkB signaling proteins (*n* = 12 (2)), cellular stress and adaptation proteins (16 (8)), cellular differentiation proteins (*n* = 11), inflammatory proteins upregulated (*n* = 13 (2)), inflammatory proteins downregulated (*n* = 17), cell protection-related proteins (*n* = 19), antioxidant-related proteins (*n* = 6), p53-mediated apoptosis proteins (*n* = 13 (2)), FAS-mediated apoptosis proteins (*n* = 9 (1)), angiogenesis-related proteins (*n* = 17 (4)), antioxidant and protection-related (11 (2)), oncogenic proteins (*n* = 12 (4)), and cytoplasmic housekeeping proteins (*n* = 3) (Table 2). All antibodies were acquired commercially and were suitable for IP and specific for proteins of mouse origin.

**Table 2:** Antibodies used in the study

Signaling proteins	No.	Antibodies
Proliferation-related proteins	11	Ki-67^*^, PCNA^a^, CDK4^a^, PLK4^a^, lamin A/C, MPM2^a^, cyclin D2, p14, p16^a^, p21^a^, p27^a^
cMyc/MAX/MAD network proteins	3	cMyc^a^, MAX^a^, MAD^a^
p53/Rb/E2F signaling proteins	5 (1)	p53, Rb-1^#^, E2F-1^a^, MDM2^a^, (CDK4)
Wnt/β-catenin signaling proteins	5	Wnt1^a^, β-catenin^a^, APC^a^, snail^a^, TCF-1^a^
Epigenetic modification proteins	6	DMAP1^a^, histone H1^a^, KDM4D^$^, HDAC-10^$^, MBD4^a^, DNMT1^a^
Protein translation proteins	5	DOHH^a^, DHS^a^, elF5A-1^$^, elF5A-2^$^, eIF2AK3^a^
Growth factor proteins	16	FGF-1^a^, FGF-2^a^, HGF-1^a^, TGF-β1^#^, TGF-β2, SMAD4^a^, IGF-1^a^, IGFIIR^a^, GH^a^, GHRH^a^, HER1^a^, HER2^a^, Erβ^a^, insulin^@^, Met^a^, CTGF^a^
RAS signaling proteins	12	NRAS^$^, KRAS^$^, STAT3^a^, SOS-1^a^, SOS-2^a^, RAF-B^a^, JNK-1^a^, ERK1^a^, p-ERK^$^, MEKK^a^, pAKT1/2/3, PI3K^a^
NFkB signaling proteins	12 (2)	NFkB^a^, IKK^a^, GADD45^a^, GADD153, MDR^a^, mTOR^@^, p38^a^, p-p38^a^, AMPK, PGC-1α, (ERK1^a^, p-ERK^a^)
Cellular stress and adaptation proteins	16 (8)	LC3^a^, PLC-β2^a^, PKC^a^, p-PKC^a^, AKAP^a^, NFAT5^a^, leptin^a^, HXK II^a^, (pAKT1/2/3^a^, p38^a^, GADD45^a^, PI3K^a^, MDM2^a^, mTOR^a^, ERK-1^a^, MDR^a^)
Inflammatory proteins upregulated	13 (2)	IL-8, IL-12^a^, CD31^a^, COX1, α1-AT, LL-37, hepcidin^a^, (TGF- β1^#^, TGF- β2), MMP-1^a^, MMP-3^a^, MMP-9^a^, MMP-10^a^
Inflammation proteins downregulated	17	TNFα^@^, IL-1^a^, IL-6^a^, IL-10^a^, IL-28^a^, M-CSF^a^, lysozyme^a^, COX-2^a^, CD56^a^, CD68^a^, CD99^a^, cathepsin C^a^, cathepsin G^a^, CRP-1^a^, lactoferrin^a^, MMP-2^a^, MMP-12^a^
Cellular differentiation proteins	11	TGase-2^$^, p63^$^, caveolin^a^, Jagged2^a^, Notch 1^a^, GLI-1^a^, Muc1^a^, Muc4^a^, AP-1^a^, SP-1^a^, SP-3^a^
Antioxidant and protection-related	11 (2)	HO-1^a^, SOD-1^a^, GST-1^a^, NOS-1^$^, LC3^a^, HSP-27^a^, HSP-70^a^, HSP-90^a^, (leptin^a^, hepcidin), NRF2^a^
p53-mediated apoptosis proteins	13 (2)	BCL2^a^, BAX^a^, BAD^a^, BAK^a^, BID^a^, AIF^a^, APAF-1^a^, caspase 9^a^, c-caspase 9^a^, PARP^a^, c-PARP^a^(p53^a^, MDM2^a^)
FAS-mediated apoptosis proteins	9 (1)	FASL^a^, FAS^a^, FADD^a^, FLIP^a^, caspase 8^a^, caspase 3^a^, c-caspase 3^a^, (BID)
Oncogenic proteins	12 (4)	PTEN^&^, DMBT-1^a^, CEA^a^, 14–3-3^a^, survivin^@^, YAP^a^ 1, TERT^a^, (pAKT1/2/3^a^, MBD4^a^, Muc1^a^, Muc4^a^), ATM^a^
Angiogenesis-related proteins	17 (4)	HIF^&^, VEGF-A^a^, VEGF-C^a^, angiogenin^$^, LYVE-1^a^, CMG2^$^, vWF^$^, FLT-4^$^, ET-1^a^, PAI-1^a^, plasminogen, PDGF-A^a^, VCAM^a^, (COX-1^a^, leptin^a^, CD31, FGF-2^a^)
Control housekeeping proteins	3	α-tubulin^a^, β-actin^a^, GAPDH^a^
Total	223 (26)	tTotal 197 antibodies

The number inside the parenthesis represents the antibodies overlapped

^a^Santa Cruz Biotechnology, USA

^#^DAKO, Denmark

^$^Neomarkers, CA, USA

^@^ZYMED, CA, USA

^&^Abcam, Cambridge, UK

Abbreviations: *α1-AT* α-1 antitrypsin; *AMPK* AMP-activated protein kinase; p-AKT1/2/3 phosphorylated (p-Akt, Thr 308); *APAF-1* apoptotic protease-activating factor 1; *AP-1* activating protein-1; *BAD* BCL2 associated death promoter; *BAK* BCL2 antagonist/killer; *BAX* BCL2-associated X; *BCL-2* B-cell leukemia/lymphoma-2; *BID* BH3 interacting-domain death agonist; *c-caspase 3* cleaved-caspase 3, caveolin; *CD*31/56/68/99 cluster of differentiation 31/56/68/99; *CDK4* cyclin-dependent kinase 4; *CEA* carcinoembryonic antigen; *CMG2* capillary morphogenesis protein 2; *COX-1* cyclooxygenase-2; *COX-2* cyclooxygenase-2; *c-PARP* cleaved-PARP (poly-ADP ribose polymerase); *CTGF* connective tissue growth factor, cyclin D2; *DMAP1* DNA methyltransferase 1-associated protein; *DMBT1* deleted in malignant brain tumors 1; *DOHH* deoxyhypusine hydroxylase; *DHS* deoxyhypusine synthase; *E2F-1* transcription factor; *eIF2AK3 (PERK)* eukaryotic translation initiation factor 2 (protein kinase R (PKR)-like endoplasmic reticulum kinase); *elF5A-1* eukaryotic translation initiation factor 5A-1; *elF5A-2* eukaryotic translation initiation factor 5A-2; *ERβ* estrogen receptor beta; *ERK* extracellular signal-regulated protein kinases; *ET-1* endothelin-1; *FAS*; CD95/Apo1; *FASL* FAS ligand; *FADD* FAS associated via death domain; *FGF-1* fibroblast growth factor-1; *FLIP* FLICE-like inhibitory protein; *FLT-4* Fms-related tyrosine kinase 4; *GADD45* growth arrest and DNA-damage-inducible 45; *GAPDH* glyceraldehyde 3-phosphate dehydrogenase; *GH* growth hormone; *GHRH* growth hormone-releasing hormone; *GST-1* glutathione S-transferase-1; *HDAC-10* hepcidin; *HIF* hypoxia inducible factor-1α; *HO-1* heme oxygenase 1; *HER2* human epidermal growth factor receptor 2; *HGF* hepatocyte growth factor; *HSP*-27/70/90 heat shock protein-27/70/90; *HXK II* hexokinase II; *IKK* IkappaB kinase, *IGF*-1, IGFIIR; IL-1/6/8/12/28 interleukin-1/6/8/12/28, Jagged2; *JNK*-1 Jun N-terminal protein kinase; *KRAS* V-Ki-ras2 Kirsten rat sarcoma viral oncogene homolog; *LC3* microtubule-associated protein 1A/1B-light chain 3, leptin; *LYVE-1* lymphatic vessel endothelial hyaluronan receptor 1, *MAX* Myc-associated factor X; *MBD4* methyl-CpG-binding domain protein 4; *M-CSF* macrophage colony-stimulating factor; *MEKK* MEK kinase; *MDM2* mouse double minute 2 homolog; *MDR* multiple drug resistance; *MPM2* mitotic protein monoclonal 2; *mTOR* mammalian target of rapamycin; *cMyc* V-myc myelocytomatosis viral oncogene homolog; *NOS-1* nitric oxide synthase 1; *NRAS* neuroblastoma RAS viral oncogene homolog, Notch 1, p16, p21, p27, p38, p53, p63, *PAI* plasminogen activator inhibitor-1; *PARP* poly-ADP ribose polymerase; *PCNA* proliferating cell nuclear antigen, *PDGF-A* platelet-derived growth factor-A; *PLC-β2* 1-phosphatidylinositol-4,5-bisphosphate phosphodiesterse β-2; *PI3K* phosphatidylinositol-3-kinases; *PLK4* polo-like kinase 4 or serine/threonine-protein kinase; *PKC* protein kinase C, *p-p38* phosphor-p38; *PTEN* phosphatase and tensin homolog; *Rb-1* retinoblastoma-1; *SMAD4* mothers against decapentaplegic; drosophila homolog 4; *SOD-1* superoxide dismutase-1; *SP-1* specificity protein 1; *STAT3* signal transducer and activator of transcription-3; *TGF-β1* transforming growth factor-β1; *TERT* human telomerase reverse transcriptase; *TNF-α* tumor necrosis factor-α, β-actin, 14-3-3; *VEGF* vascular endothelial growth factor; *VCAM* vascular cell adhesion, *vWF* Von Willebrand factor; *YAP 1* yes-associated protein

Briefly, protein samples were mixed with 5 mL of binding buffer (150 mM NaCl, 10 mM Tris-HCl pH 7.4, 1 mM EDTA, 1 mM EGTA, 0.2 mM sodium vanadate, 0.2 mM phenylmethylsulfonyl fluoride (PMSF) and 0.5% Tergitol-type NP-40 (nonyl phenoxypolyethoxylethanol) and incubated in protein A/G agarose columns at 4 °C for 1 h (columns were placed on a rotating stirrer during the incubation). After washing each column with sufficient PBS (phosphate-buffered saline), target proteins were eluted using 150 μL of IgG elution buffer (Pierce, USA). Immunoprecipitated proteins were analyzed using a HPLC unit (1100 series, Agilent, USA) equipped with a reverse phase column and a micro-analytical detector system (SG Highteco, South Korea). Elution was performed using 0.15 M NaCl containing 20% acetonitrile at 0.4 mL/min for 30 min, and detection by UV spectroscopy at 280 nm. Control and experimental samples were run sequentially to allow comparisons. For IP-HPLC, whole protein peak areas (mAU*s) were calculated by subtracting the antibody peak areas of negative controls, and experimental protein peak area square roots were compared with control one (Additional file 2) [16].

When IP-HPLC results were compared with western blot data for cytoplasmic housekeeping protein (β-actin), IP-HPLC errors were < ± 5%, whereas western blot errors exceeded ± 20% and were not suitable for statistical analysis (Additional file 3) [16]. In particular, repeat IP-HPLC runs (4–10 runs) to determine errors associated with protein expression revealed errors were ± 5% (Additional file 4) [16]. Based on these findings, IP-HPLC was used rather than western blot to analyze protein expressional changes.

### Statistical analysis

Proportions (%) of experimental and control groups were plotted (Additional file 4), and the analysis was repeated two to six times (until mean and standard deviations were ≤ ± 5%). Results were analyzed using the chi-squared test [18–20]. The expression of control housekeeping proteins, that is, *β*-actin, *α*-tubulin, and GAPDH (glyceraldehyde 3-phosphate dehydrogenase), were relatively unchanged (≤ 5%) by DCE-2.5, 5, or 10. Protein expression changes were defined as follows: non-significant for ≤ ± 5%, slight for ± 5–10%, marked for ± 10–20%, and great for ≥ ± 20%.

## Results

### Histological observations

**Fig. 1:**
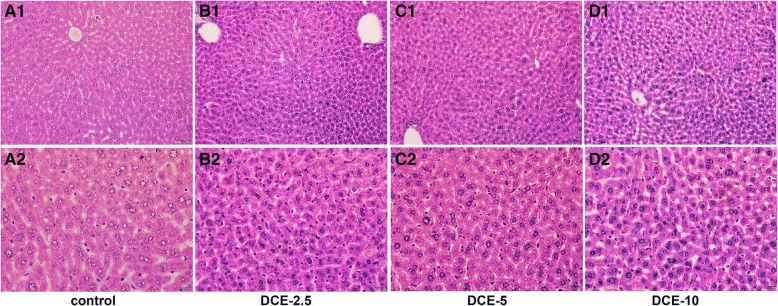
Photomicrographs of mouse liver, hematoxylin and eosin stain. **a** Normal mouse liver. **b** Mouse liver treated with DCE-2.5, denoting hypertrophic hepatocytes with increased nuclear heterochromatism. **c** Mouse liver treated with DCE-5 had hypertrophic hepatocytes, which formed thick hepatic cords. **d** In the livers of mice treated with DCE-10, hepatocytes were slightly shrunken and aligned, and sinusoidal spaces were enlarged

Mouse livers treated with DCE-2.5 showed hypertrophic hepatocytes containing more eosinophilic cytoplasms than non-treated controls. Most hepatic cords were thickened with hypertrophic hepatocytes, but their sinusoidal spaces were well preserved. Many hepatocytes showed increased heterochromatic nuclei, but no necrotic hepatocytes were observed during the histological observation (Fig. 1b). Mouse livers treated with DCE-5 consistently showed hypertrophic hepatocytes and narrow sinusoidal spaces (Fig. 1c), while those treated with DCE-10 showed shrunken hepatocytes and had larger sinusoidal spaces than non-treated controls (Fig. 1d).

### Immunohistochemical observation

**Fig. 2:**
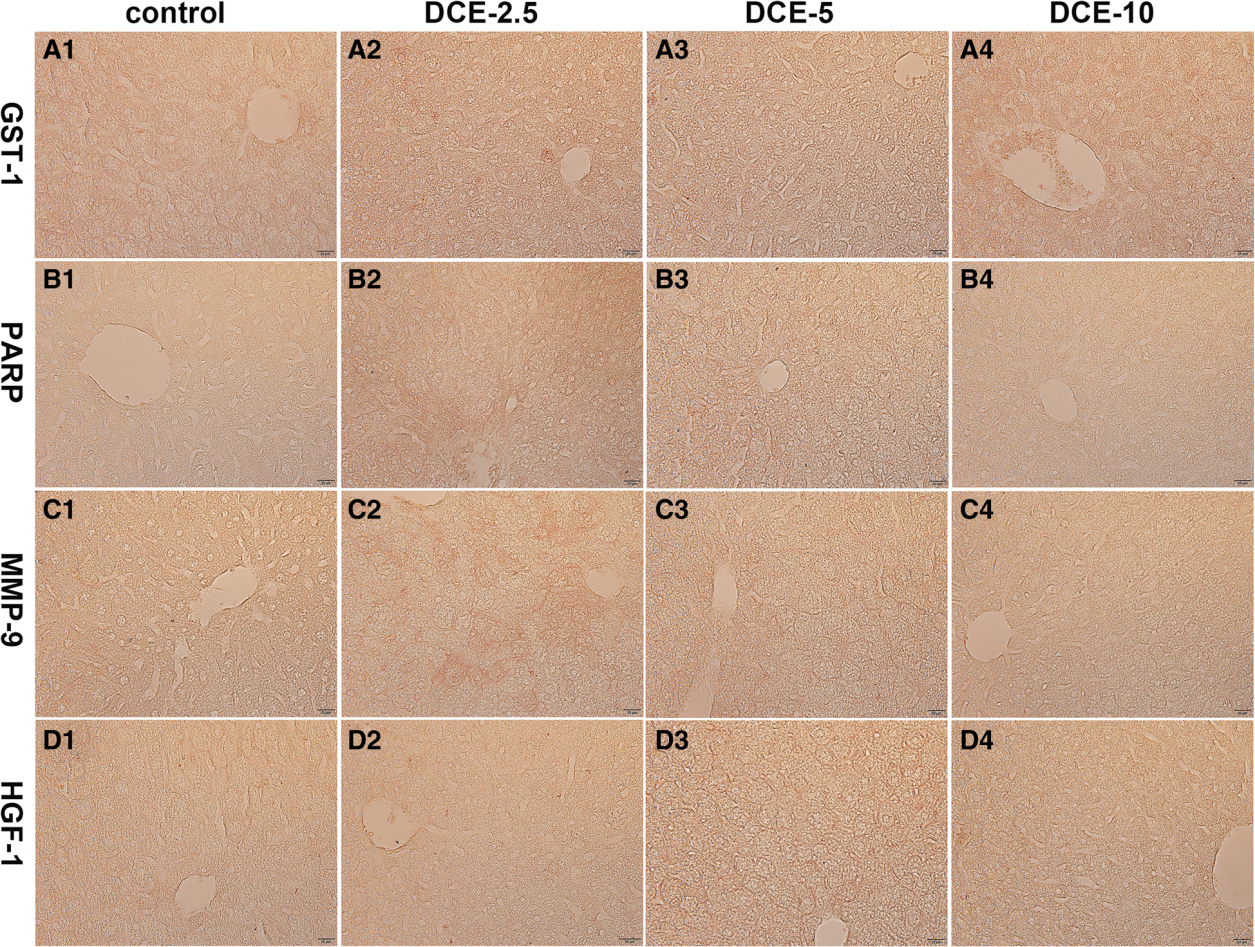
Photomicrographs of mouse liver, immunohistochemical staining for GST-1, PARP, MMP-9, and HGF-1. **a** GST-1 expression was increased dose-dependently by DCE-2.5 to DCE-10. **b** PARP was markedly positive in the DCE-2.5 group. **c** MMP-9 was strongly positive in the DCE-2.5 group. **d** HGF-1 was gradually and dose-dependently increased by DCE

Representative antibodies of GST-1, PARP, MMP-9, and HGF-1 were applied to mouse liver sections. Hepatocyte regeneration and expressions of protective proteins, HGF-1 and GST-1, were dose-dependently increased in mouse livers treated with DCE-2.5, DCE-5, or DCE-10. HGF-1 was strongly expressed at hepatocyte membranes in the DCE-5 group, while GST-1 was strongly positive in hepatocyte cytoplasms in the DCE-10 group (Fig. 2A1–A4, B1–B4). Mouse livers treated with DCE-2.5, DCE-5, or DCE-10 were positive for PARP and MMP-9 (cellular apoptosis and scavenging proteins, respectively). Both PARP and MMP-9 were strongly positive in the DCE-2.5 group, and consistently positive in the DCE-5 and DCE-10 groups (Fig. 2C1–C4, D1–D4).

### Effects of DCE on the expressions of proliferation-related proteins in mouse livers

**Fig. 3:**
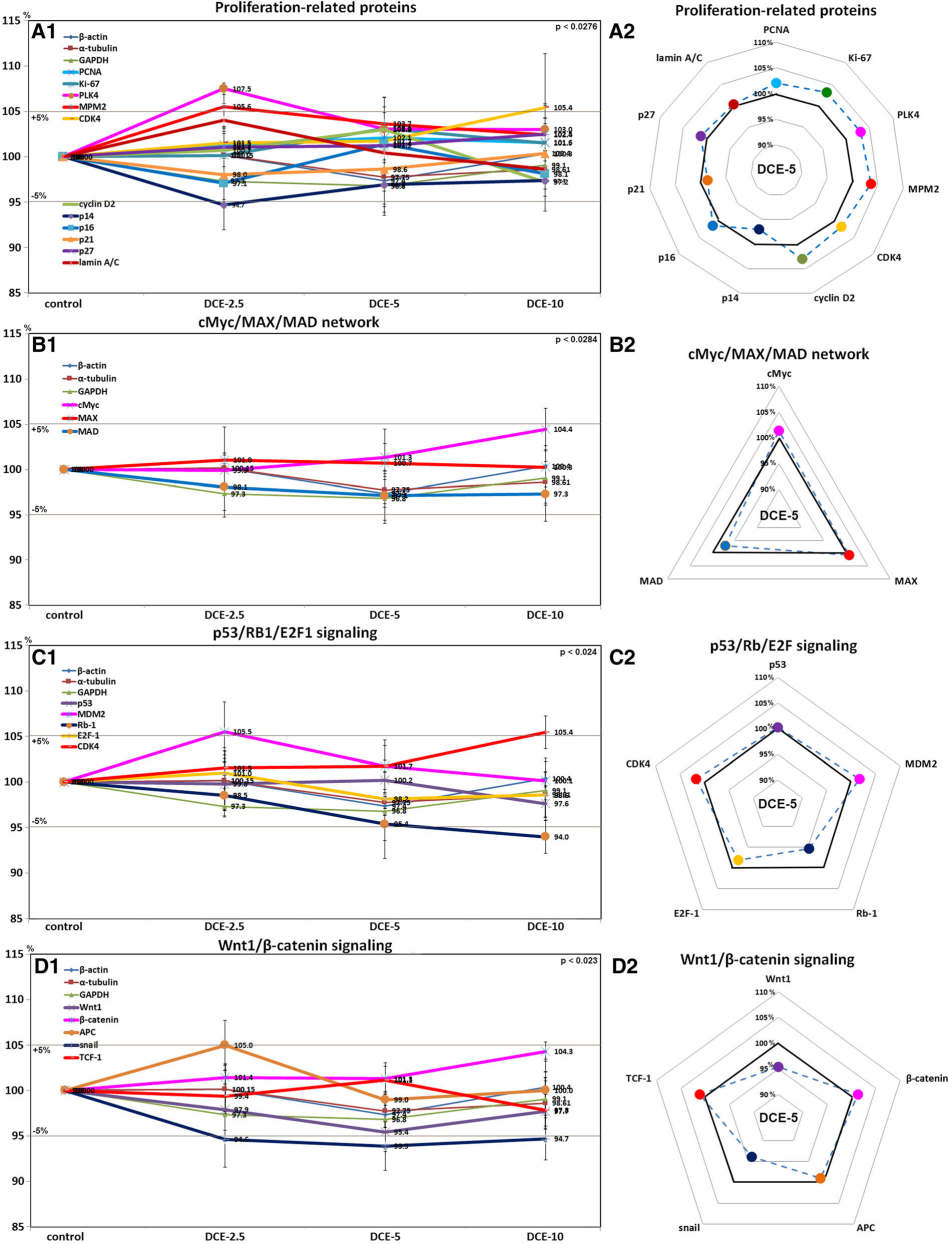
Expression levels of proliferation-related proteins (**a**), cMyc/MAX/MAD signaling proteins (**b**), p53/Rb/E2F signaling proteins (**c**), and Wnt1/β-catenin signaling proteins (**d**) after DCE treatment in mouse livers as determined by IP-HPLC. Major protein expression pattern induced by DCE-5 was found in the radial graphs of A2, B2, C2, and D2

Mouse livers treated with DCE-2.5 or DCE-5 showed higher expressions of proliferation-related proteins (PLK4 (107.5%) and MPM2, (105.6%)) but lower p14 expression (94.7%) than non-treated controls. The expression levels of other proliferation-related proteins, PCNA, Ki-67, CDK4, cyclin D2, p16, p21, p27, and lamin A/C like those of the control housekeeping proteins (β-actin, α-tubulin, and glyceraldehyde-3-phosphate dehydrogenase, GAPDH) changed by less than ± 5% in response to DCE (Fig. 3A1). However, PCNA, Ki-67, CDK4, cyclin D2, and lamin A/C were upregulated; and p16, p21, and p27 were downregulated dose-dependently by DCE (Fig. 3A2). These results suggested that DCE-2.5 and DCE-5 slightly enhanced the proliferation of murine hepatocytes.

### Effects of DCE on the expressions of cMyc/MAX/MAD network proteins in mouse livers

Mouse livers treated with DCE showed expression changes of cMyc, MAX, and MAD of < ± 5% as was observed for control housekeeping proteins (Fig. 3B1, B2), indicating DCE did not activate cMyc/MAX signaling in murine hepatocytes.

### Effects of DCE on the expressions of p53/Rb/E2F signaling proteins in mouse livers

DCE-2.5 or DCE-5 altered the expressions of p53, MDM2, Rb-1, E2F-1, and CDK4 by < ± 5%, which was similar to those observed for control housekeeping proteins, but DCE-10 increased the expression of CDK4 (105.4%) and reduced the expression of Rb-1 (94%) as compared with non-treated control livers (Fig. 3C1, C2). These results suggest that DCE-2.5 and DCE-5 did not activate p53/Rb/E2F signaling in murine hepatocytes.

### Effects of DCE on the expression of Wnt1/β-catenin signaling proteins in mouse livers

Mouse livers treated with DCE showed expression changes in Wnt1, β-catenin, APC, and TCF-1 of < ± 5%, which was similar to that observed for control housekeeping proteins, but had reduced snail levels (93.9%) (Fig. 3D1, D2). These results suggested DCE did not activate Wnt/β-catenin signaling in murine hepatocytes.

### Effects of DCE on the expressions of epigenetic modification-related proteins in mouse livers

**Fig. 4:**
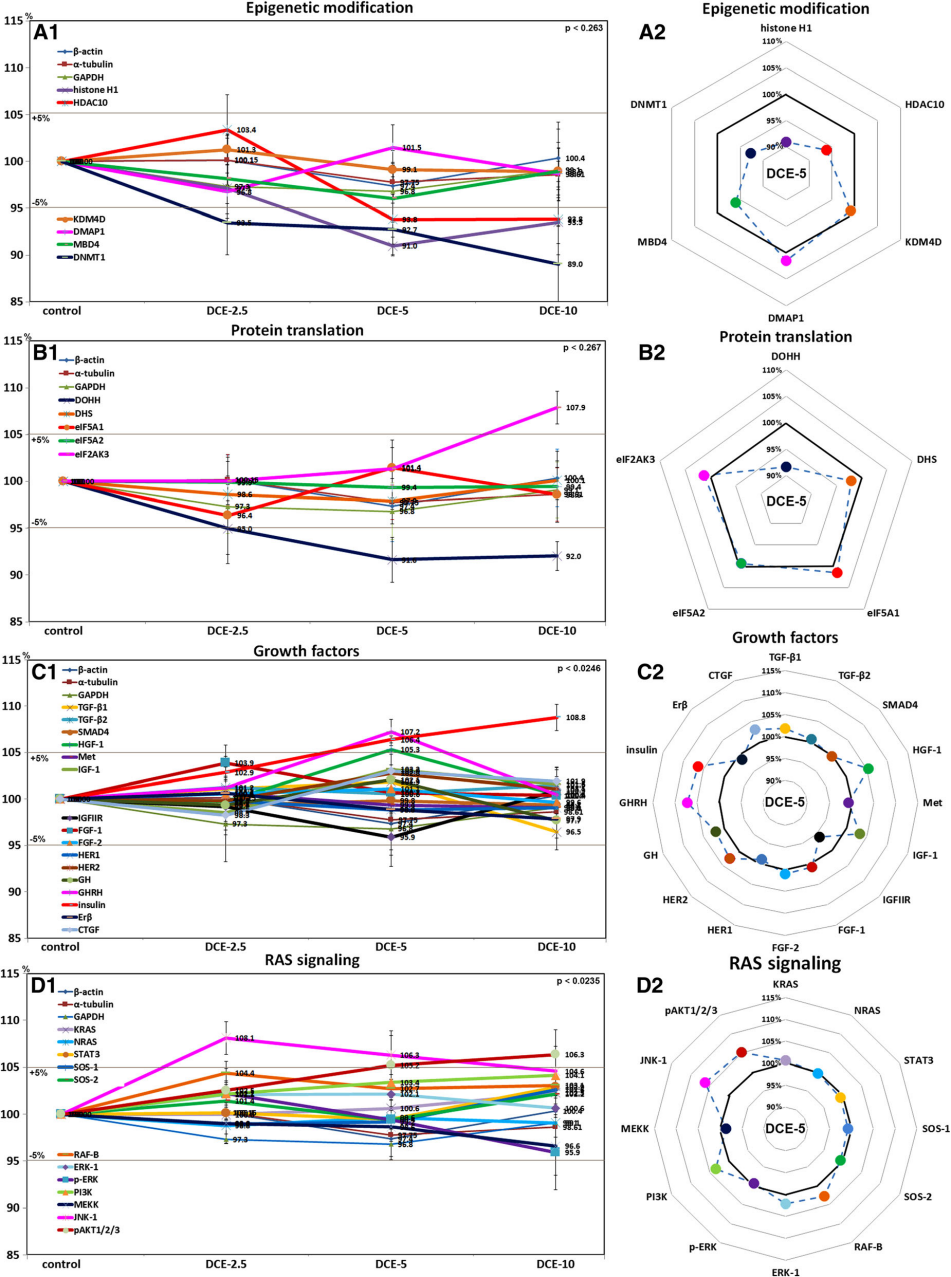
Expressions of epigenetic modification-related proteins (**a**), protein translation-related proteins (**b**), growth factors (**c**), and RAS signaling proteins (**d**) after DCE treatment in mouse livers as determined by IP-HPLC. Major protein expression pattern induced by DCE-5 was found in the radial graphs of A2, B2, C2, and D2

Mouse livers treated with DCE showed DMAP1, KDM4D, and MBD4 level changes of < ± 5%, as was observed for control housekeeping proteins, but the expressions of histone H1, HDAC10, and DNMT1 reduced to 91%, 93.8%, and 89%, respectively, after treatment with DCE-5 or DCE-10 (Fig. 4A1, A2). These results suggested DCE slightly activated epigenetic modification by downregulating histone H1, HDAC10, and DNMT1.

### Effects of DCE on the expressions of protein translation-related proteins in mouse livers

Mouse livers treated with DCE showed expression changes of DHS, eIF5A-1, eIF5A-2, and eIF2AK3 of < ± 5%, like control housekeeping proteins, but showed slight decreases in the levels of DOHH (91.6%) after treatment with DCE-5 or DCE-10, and a slight increase in eIF2AK3 (107.9%) after treatment with DCE-10 (Fig. 4B1, B2). These results suggested DCE slightly inactivated protein translation by downregulating DOHH and upregulating eIF2AK3.

### Effects of DCE on levels of growth factor proteins in mouse livers

Mouse livers treated with DCE-5 showed higher expressions of GHRH (107.2%), HGF-1 (105.3%), and insulin (106.4%) than non-treated controls, and those treated with DCE-10 showed higher insulin levels (108.8%). The expression levels of other growth factors, TGF-β1, TGF-β2, SMAD4, Met, IGF-1, IGFIIR, FGF-1, FGF-2, HER1, HER2, GH, Erβ, and CTGF changed by < ± 5%, as was observed for control housekeeping proteins (Fig. 4C1). However, protein levels of IGF-1, FGF-2, GH, and CTGF were upregulated and of FGF-1 and Erβ were downregulated by DCE-5 (Fig. 4C2). These results suggest DCE-2.5 and DCE-5 slightly increased growth factor levels in murine hepatocytes.

### Effects of DCE on the expressions of RAS signaling proteins in mouse livers

Mouse livers treated with DCE-5 showed higher expressions of JNK-1 (106.3%), and those treated with DCE-10 showed higher expression of pAKT1/2/3 (106.3%) than non-treated controls. The expression levels of other RAS signaling proteins, KRAS, NRAS, STAT3, SOS-1, SOS-2, RAF-B, ERK-1, p-ERK-1, PI3K, and MEKK changed by < ± 5% in response to DCE as was observed for control housekeeping proteins (Fig. 4D1), but DCE-5 increased the levels of RAF-B, ERK-1, PI3K, JNK-1, pAKT1/2/3 (Fig. 4D2). These results suggested that DCE-2.5 and DCE-5 slightly enhanced RAS signaling in murine hepatocytes.

### Effects of DCE on levels of NFkB signaling proteins in mouse livers

**Fig. 5:**
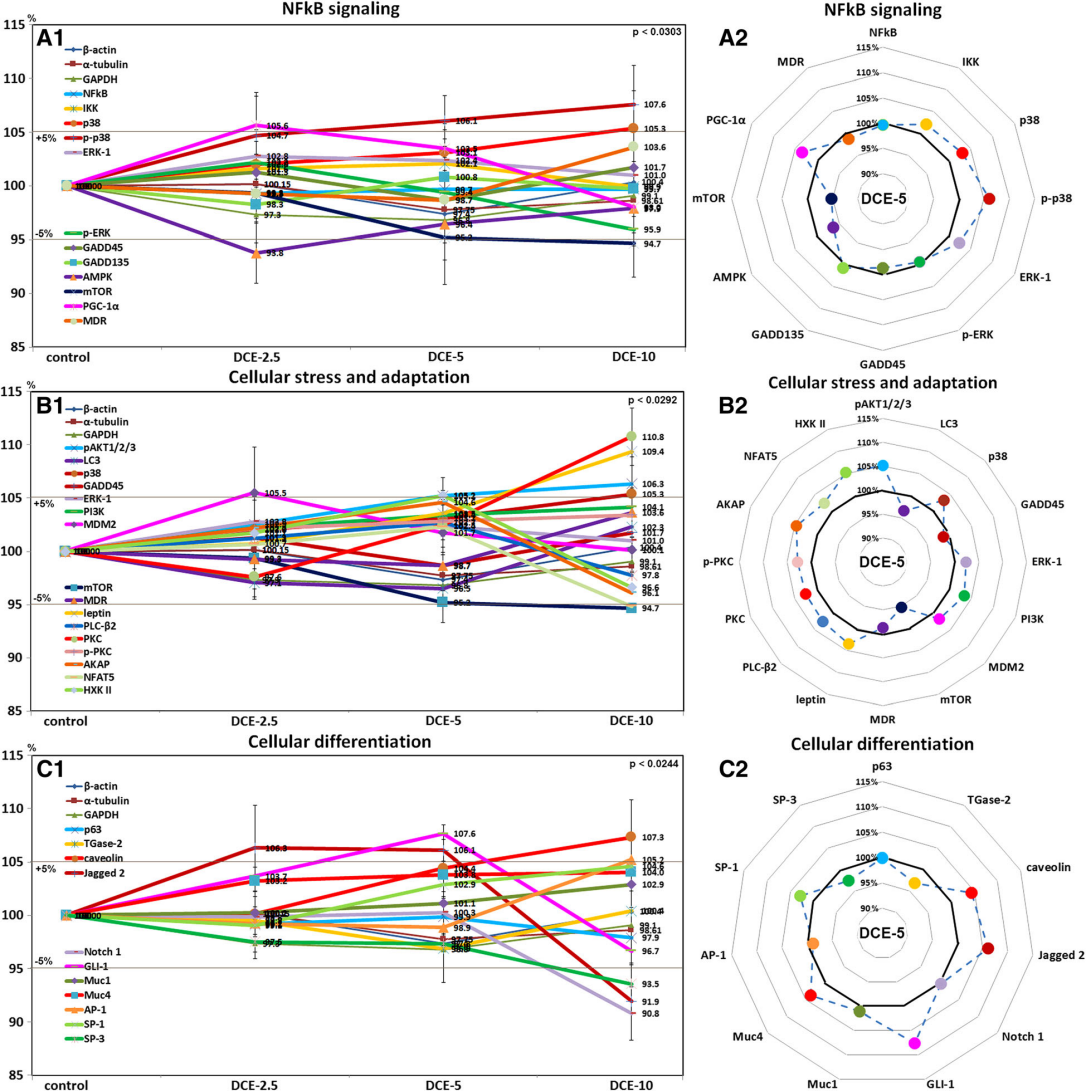
Expressions of NFkB signaling (**a**), cellular stress and adaptation (**b**), and cellular differentiation (**c**) protein levels after DCE treatment in mouse livers as determined by IP-HPLC. Major protein expression pattern induced by DCE-5 was found in the radial graphs of A2, B2, C2, and D2

Mouse livers treated with DCE-2.5 showed higher PGC-1α (105.6%) and lower AMPK (93.8%) than non-treated controls, whereas those treated with DCE-5 and DCE-10 showed higher p-38 (107.6%) levels and lower mTOR (94.7%) levels. The levels of other NFkB signaling proteins, that is, NFkB, IKK, ERK-1, p-ERK, GADD45, GADD153, and MDR changed by < ± 5% in response to DCE as was observed for control housekeeping proteins (Fig. 5A1), but DCE-5 increased the level of IKK, p38, p-p38, ERK-1, and PGC-1α (Fig. 5A2). These results suggested DCE-2.5 and DCE-5 slightly inhibited NFkB signaling in murine hepatocytes.

### Effects of DCE on cellular stress and adaptation-related protein levels in mouse livers

Mouse livers treated with DCE-2.5 showed a slight increase in MDM2 (105.5%), whereas those treated with DCE-5 or DCE-10 showed higher PKC (110.8%), leptin (109.4%), p38 (105.3%), and pAKT1/2/3 (106.3%) levels and lower mTOR (94.7%) levels than non-treated controls. The expression levels of other cellular stress and adaptation-related proteins, that is, LC3, GADD45, ERK-1, MDR, PLC-β2, AKAP, NFAT5, and HXK II changed by < ± 5% in response to DCE, as was observed for control housekeeping proteins (Fig. 5B1), but DCE-5 tended to increase p38, ERK-1, PI3K, leptin, PLC-β2, PKC, p-PKC, AKAP, NFAT5, and HXK II levels (Fig. 5B2). These results suggested that DCE-2.5 and DCE-5 slightly enhance cellular stress and adaptation-related protein levels in murine hepatocytes.

### Effects of DCE on cellular differentiation-related protein levels in mouse livers

Mouse livers treated with DCE-2.5 and DCE-5 showed higher Jagged 2 (106.3%) and GLI-1 (107.6%) levels, whereas those treated with DCE-10 showed higher caveolin (107.3%) and lower SP-3 (93.5%), Notch 1 (90.8%), and Jagged 2 (91.9%) levels than non-treated controls. The expression levels of other cellular differentiation-related proteins, p63, TGase-2, Muc1, Muc4, AP-1, and SP-1 changed by < ± 5% in response to DCE, as was observed for control housekeeping proteins (β-actin, α-tubulin, and glyceraldehyde-3-phosphate dehydrogenase, GAPDH) (Fig. 5C1), but DCE-5 tended to increase caveolin, Jagged 2, Notch 1, GLI-1, Muc4, and SP-1 levels (Fig. 5C2). These results suggested DCE-2.5 and DCE-5 slightly enhance the expressions of cellular differentiation-related proteins in murine hepatocytes.

### Effects of DCE on inflammatory proteins upregulated in mouse livers

**Fig. 6:**
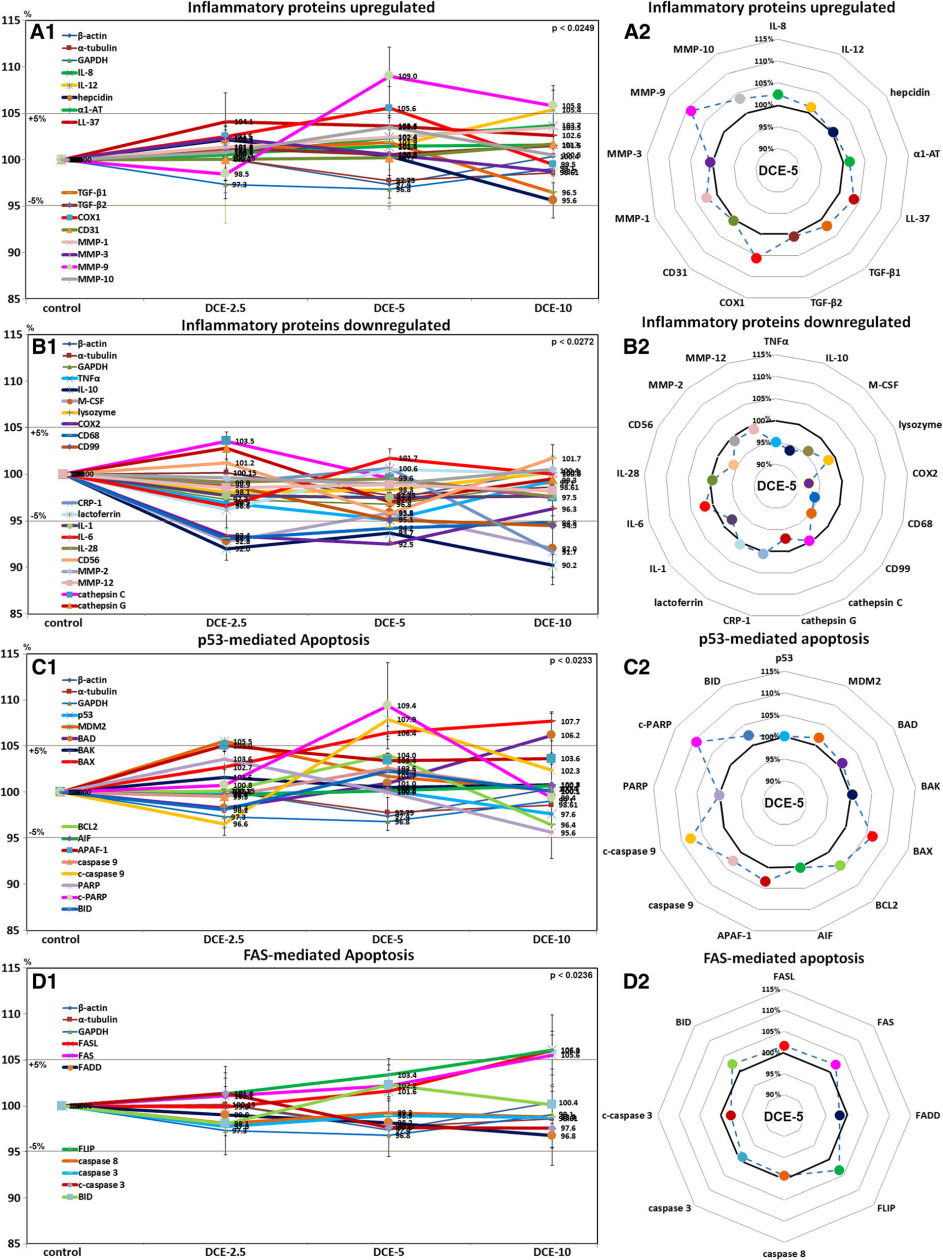
Expressions of inflammatory proteins upregulated (**a**), inflammatory proteins downregulated (**b**), p53-mediated apoptosis proteins (**c**), and FAS-mediated apoptosis proteins (**d**) after DCE treatment in mouse livers as determined by IP-HPLC. Major protein expression pattern induced by DCE-5 was found in the radial graphs of A2, B2, C2, and D2

Mouse livers treated with DCE-5 and DCE-10 showed higher MMP-9 (109%), COX-1 (105.6%), and IL-12 (105.4%) levels than non-treated controls. The expression levels of other inflammatory proteins, that is, IL-8, hepcidin, α1-AT, LL-37, TGF-β1, TGF-β2, CD31, MMP-1, MMP-3, and MMP-10 were upregulated by < ± 5% in response to DCE, as was observed for control housekeeping proteins (Fig. 6A1). However, LL-37, COX-1, and MMP-10 tended to be upregulated by DCE-5 as compared with housekeeping controls (Fig. 6A2). These results suggested DCE-5 and DCE-10 tended to increase the expressions of inflammatory proteins for cellular regeneration in mouse livers.

### Effects of DCE on inflammatory proteins downregulated in mouse livers

TNFα (95.1%), IL-10 (90.2%), COX-2 (92.5%), CD68 (93.1%), M-CSF (92%), and CRP-1 (91.7%) were lower in DCE-treated mouse livers than in non-treated controls. Levels of other inflammatory proteins, that is, lysozyme, CD99, lactoferrin, IL-1, IL-6, IL-28, CD56, MMP-2, MMP-12, cathepsin C, and cathepsin G were changed by < ± 5% in response to DCE, as was observed for control housekeeping proteins (Fig. 6B1), but DCE-5 tended to reduce TNFα, IL-10, M-CSF, COX-2, CD68, CD99, cathepsin G, IL-1, and CD56 levels (Fig. 6B2). These results suggested DCE-2.5 and DCE-5 reduced the expressions of inflammatory proteins eliciting inflammatory reaction in mouse livers.

### Effects of DCE on p53-mediated apoptosis-related protein levels in mouse livers

Mouse livers treated with DCE-5 or DCE-10 showed higher expressions of BAX (107.7%), BAD (106.2%), APAF-1 (105%), c-PARP (109.4%), and c-caspase 9 (107.9%) than non-treated controls. The expression levels of other p53-mediated apoptosis-related proteins, p53, MDM2, BAK, BCL2, AIF, and BID changed by < ± 5% in response to DCE, as was observed for control housekeeping proteins (Fig. 6C1), but DCE-5 tended to increase the levels of BAX, BCL2, APAF-1, c-caspase-9, and c-PARP (Fig. 6C2). These results suggested that DCE-5 and DCE-10 slightly enhance the expressions of p53-mediated apoptosis-related proteins in murine hepatocytes.

### Effects of DCE on FAS-mediated apoptosis-related protein levels in mouse livers

Mouse livers treated with DCE-10 had higher FASL (106.1%), FAS (105.6%), and FLIP (106%) levels than non-treated controls. The levels of other FAS-mediated apoptosis-related proteins, that is, FADD, caspase 8, caspase 3, c-caspase 3, and BID, changed by < ± 5% in response to DCE, as was observed for control housekeeping proteins (Fig. 6D1, D2). These results suggested that DCE-2.5 and DCE-5 did not induce FAS-mediated apoptosis-related protein expressions in murine hepatocytes.

### Effects of DCE on angiogenesis-related protein levels in mouse livers

**Fig. 7:**
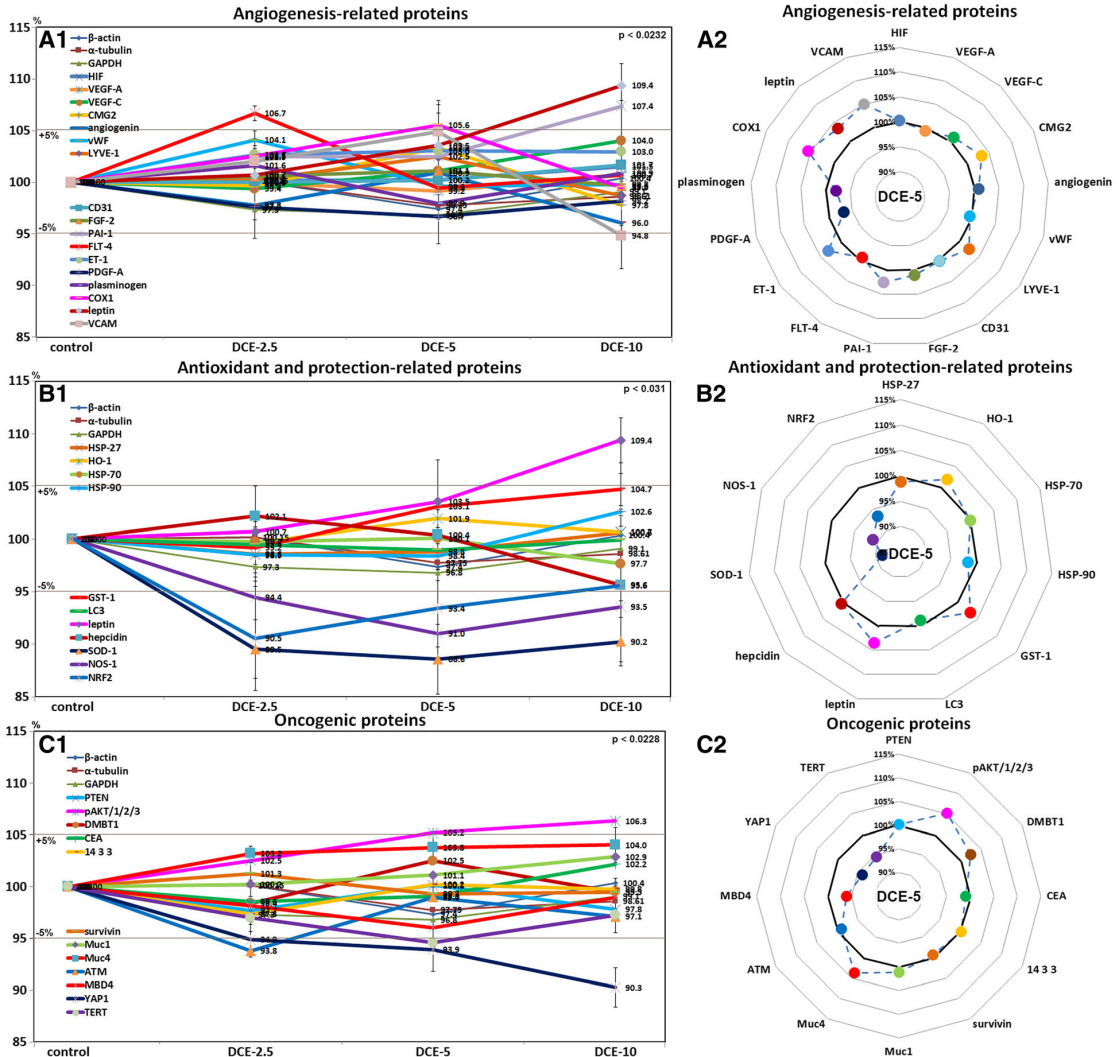
Expressions of angiogenesis-related proteins (**a**), antioxidant and protection-related proteins (**b**), and oncogenic proteins (**c**) after DCE treatments of mouse livers as determined by IP-HPLC. Major protein expression pattern induced by DCE-5 was found in the radial graphs of A2, B2, C2, and D2

Mouse livers treated with DCE-5 or DCE-10 had higher FLT-4 (106.7%) and COX-1 (105.6%) levels, and those treated with DCE-10 had higher leptin (109.4%) and PAI-1 (107.4%) levels but lower VCAM (94.8%) levels than non-treated controls. The expression levels of other angiogenesis-related proteins, that is, of HIF, VEGF-A, VEGF-C, CMG2, angiogenin, vWF, LYVE-1, CD31, FGF-2, ET-1, and PDGF-A changed by < ± 5% in response to DCE, as was observed for housekeeping controls (Fig. 7A1), but DCE-5 tended to increase CMG2, LYVE-1, PAI-1, ET-1, COX-1, leptin, and VCAM levels (Fig. 7A2). These results suggested DCE-2.5 and DCE-5 slightly enhanced the expressions of angiogenesis-related proteins in mouse livers.

### Effects of DCE on antioxidant and protection-related protein levels in mouse livers

Mouse livers treated with DCE-2.5 or DCE-5 had lower NRF2 (90.5%), NOS-1 (91%), and SOD-1 (88.6%) levels than non-treated controls and those treated with DCE-10 had higher leptin (109.4%) levels but consistently lower NRF2 (95.6%), NOS-1 (93.5%), and SOD-1 (90.2%) levels than non-treated controls. The expression levels of other antioxidant and protection-related proteins, that is, HSP-27, HSP-70, HSP-90, HO-1, GST-1, LC3, and hepcidin, changed by < ± 5% in response to DCE, as was observed for housekeeping controls (Fig. 7B1), but DCE-5 tended to increase HO-1, GST-1, and leptin levels (Fig. 7B2). These results suggested DCE-2.5 and DCE-5 reduced free radical concentrations in mouse livers and subsequently reduced antioxidant-related protein levels, but did not affect the expressions of cellular protection-related proteins in murine hepatocytes.

### Effects of DCE on oncogenic protein levels in mouse livers

Mouse livers treated with DCE had lower YAP1 (90.3%), ATM (93.8%), and TERT (94.6%) levels, but slightly higher pAKT1/2/3 (106.3%) levels than non-treated controls. The expression levels of other oncogenic proteins, that is, PTEN, DMBT1, CEA, 14-3-3, survivin, Muc1, Muc4, and MBD4 changed by < ± 5% in response to DCE, as was observed for control housekeeping proteins (Fig. 7C1), but DCE-5 tended to reduce CEA, MBD4, YAP1, and TERT levels (Fig.7C2). These results suggested DCE slightly reduced the expressions of oncogenic proteins in murine hepatocytes.

### Global protein expressions in mouse livers treated with DCE-5

Global protein expressions induced by DCE in mouse liver changed by < ± 10%, that is, they probably remained in the physiological homeostatic range. The levels of many proteins essential for molecular signaling were altered by ± 5% as was observed for housekeeping controls (β-actin, α-tubulin, and glyceraldehyde-3-phosphate dehydrogenase, GAPDH). These results indicated DCE treatment caused minimal cellular stress and did not induce inflammatory or chemical stress or oncogenic injury.

**Fig. 8:**
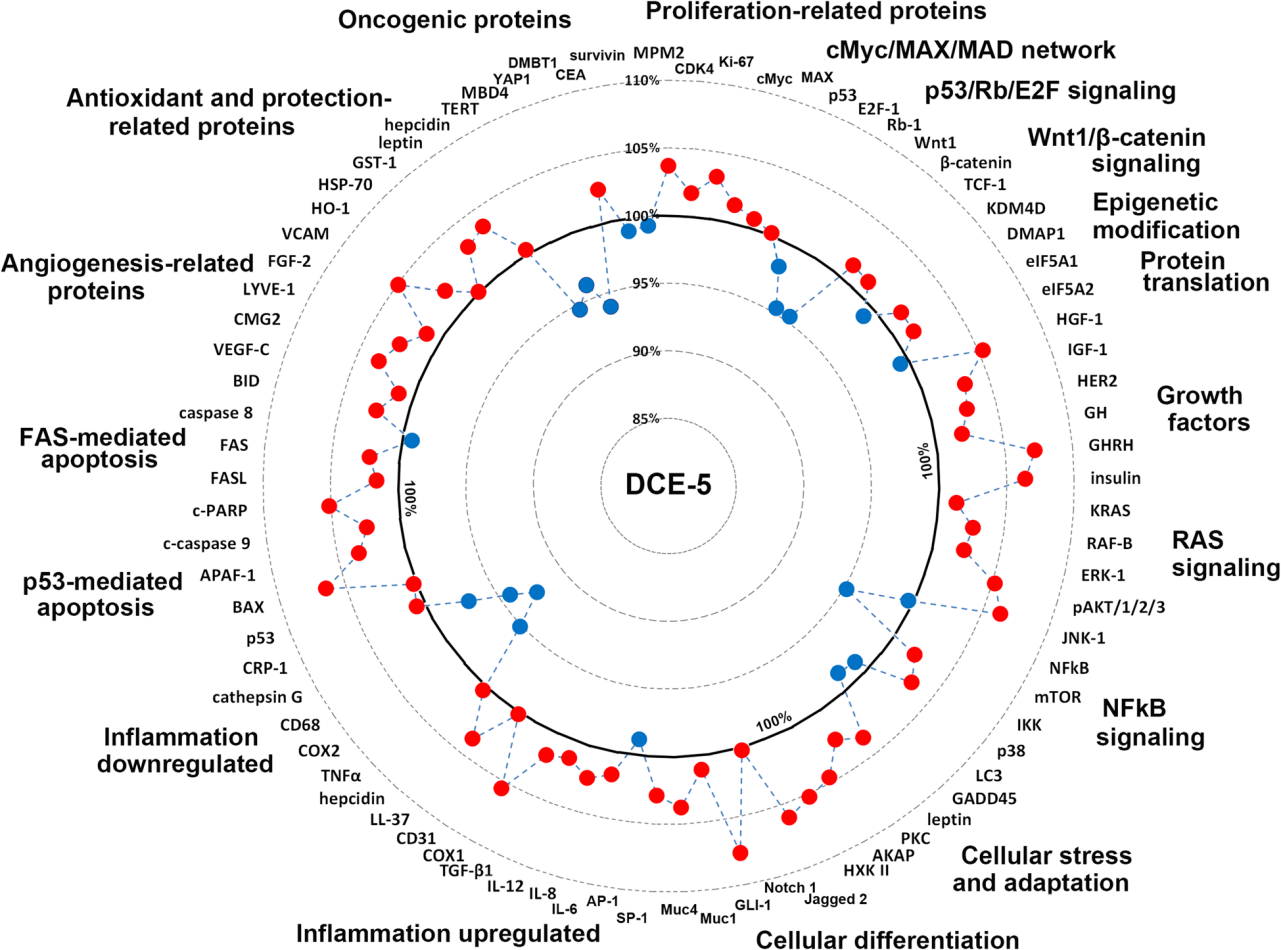
Global protein expression diagrams showing the effects of DCE-5 in mouse liver. Mouse livers treated with DCE-5 showed characteristic patterns of molecular signaling for up- (red round circles) and downregulating (blue round circles) essential proteins for cellular proliferation, protection, adaptation, differentiation, apoptosis, and regeneration

Generally, mouse livers treated with DCE-5 showed characteristic changes in functional protein levels (Fig. 8). Increases in the 10% range were observed for proliferation-related proteins (MPM2, CDK4, and Ki-67), growth factors (HGF-1, IGF-1, HER2, GH, GHRH, insulin, KRAS, RAF-B, ERK-1, pAKT1/2/3, and JNK-1), cellular adaptation-related proteins (leptin, PKC, AKAP, and HXK II), cellular differentiation-related proteins (Jagged 2, Notch 1, GLI-1, Muc1, Muc4, and SP-1), inflammatory proteins (IL-6, IL-8, IL-12, TGF-β1, COX-1, and LL-37), p53-mediated apoptosis-related proteins (BAX, APAF-1, c-caspase 9, and c-PARP), angiogenesis-related proteins (VEGF-C, CMG2, LYVE-1, FGF-2, and VCAM), and antioxidant and protection-related proteins (HO-1, HSP-70, GST-1, leptin, and hepcidin). On the other hand, decreases in the 10% range were observed for NFkB signaling proteins (mTOR, LC3, and GADD45), inflammatory proteins (TNFα, COX-2, CD68, and cathepsin G), and oncogenic proteins (TERT, MBD4, and YAP1). cMyc/MAX/MAD network proteins, p53/Rb/E2F signaling proteins, Wnt/β-catenin signaling proteins, epigenetic modification-related proteins, protein translation-related proteins, and FAS-mediated apoptosis-related proteins were rarely affected by DCE treatment (Fig. 8).

## Discussion

2.5, 5, or 10 cups of coffee are slowly absorbed through the gastrointestinal tract, whereas in the present study, DCE-2.5, DCE-5, and DCE-10 were injected intraperitoneally, which is likely to have a far greater effect on liver. However, the DCE-induced hypertrophic effect on murine hepatocytes observed was more marked than was expected, although hypertrophic hepatocytes were well aligned in hepatic cords and sinusoidal spaces were well maintained. Furthermore, no necrotic hepatocytes recruiting inflammatory cells were observed histologically, and thus, we considered the pharmacological effects of DCE-10 still lay in the homeostatic range of hepatocyte metabolism, and that DCE-2.5 which induced mild hypertrophic effect on murine hepatocytes would be safer than DCE-5 which induced marked hypertrophic effect.

Nevertheless, mice treated with DCE-10 showed marked hepatocyte shrinkage and greater sinusoidal spaces, indicating that DCE-10 caused a certain amount of cellular damage. Although no necrotic hepatocytes were observed in DCE-10-treated mouse livers, it is possible that the metabolic statuses of hepatocytes may have been diminished due to smaller amounts of hepatocyte cytoplasm observed in DCE-10 than in DCE-2.5- and DCE-5-treated mouse livers. These observations suggest that mouse livers were over-stimulated by DCE-10, and that hepatocytes underwent transient retrogressive degeneration.

The protective and antioxidant effects of DCE observed in mouse livers were similar to those reported for kahweol (a coffee-specific diterpene) in SH-SY5Y cells, which upregulated HO-1 and p38 levels [21, 22]. It has been suggested the anti-apoptotic effect of DCE in mouse liver might play a role in the radiation-protective effect of caffeine via the downregulation of BAX protein [23]. In other studies, the anti-inflammatory effects of DCE-2.5 and DCE-5 were found to be closely related to their downregulation of NFkB signaling [8, 24]. Furthermore, kahweol has been reported to suppress the proliferation and induce the apoptosis of human colorectal cancer cells, and head and neck squamous cell carcinoma cells [25–27]. However, in the present study, DCE slightly induced the proliferation of murine hepatocytes and simultaneously reduced the expressions of inflammatory proteins (TNFα, COX-2, IL-6, and CD68) and of oncogenic proteins (YAP1 and TERT).

The liver is for detoxifying various metabolites and producing biochemicals required for digestion, and its roles also include the regulation of glycogen storage, the decomposition of red blood cells, and the production of hormones. The liver is the only visceral organ that possesses the capacity to regenerate, for example, after surgical removal or chemical injury, in fact, as little as 25% of original liver mass can regenerate a full-sized liver [28, 29]. In the present study, DCE slightly increased the expressions of p53-mediated apoptosis-related proteins, but did not affect the expressions of FAS-mediated apoptosis-related proteins, and these protein expressional changes occurred in parallel with increases in the expressions of MMPs and enhanced hepatocyte proliferation. These observations suggest that DCE induced the apoptosis of old hepatocytes and enhanced scavenging of resulting debris by MMPs and hepatocyte regeneration by activating proliferation-related proteins, and followed by reactive de novo angiogenesis for sinusoidal vasculature.

The anti-inflammatory effects of DCE observed in mouse liver in the present study were consistent with its strong antioxidant effect, its inactivation of NFkB signaling, and its promotion of anti-oncogenic signaling, but the observed increase in the hepatocyte proliferation may not have been related to cMyc/MAX signaling, Rb/E2F signaling, or epigenetic modification, but rather to slight increases in the expressions of growth factors, such as HGF, IGF-1, GH, GHRH, and CTGF; and slight activation of RAS signaling involving RAF-B, ERK-1, PI3K, JNK-1, and pAKT1/2/3. In particular, DCE downregulated FGF-1, TGF-β1, TGF-β2, and SMAD4 levels (fibrosis-related proteins), but upregulated levels of Erβ and insulin (anti-fibrosis proteins) in mouse liver. These findings suggest that mouse livers treated with DCE might progressively regenerate via growth factor/RAS signaling and anti-inflammation, low cellular stress, and anti-oncogenic signaling.

Tea and coffee have been associated, both positively and negatively, with the risk of cardiovascular disease (CVD). The effects of coffee remain controversial and concerns have been expressed regarding associations between its consumption and hypercholesterolemia, hypertension, and myocardial infarction [30]. Caffeine and kahweol are known anti-angiogenic compounds [31, 32] that may function as anti-tumor and anti-myocardial infarct agents. In the present study, DCE slightly upregulated angiogenesis-related protein levels (FLT-4, COX-1, leptin, VCAM, and PAI-1) in mouse livers, which contrasts with its antiangiogenic effect in RAW 264.7 cells [16]. However, we observed these expressions of angiogenesis stimulating proteins were not associated with major angiogenesis signaling involving HIF, VEGF-A, VEGF-C, angiogenin, vWF, CD31, and PDGF-A, and thus, it would appear that the angiogenic effect of DCE in mouse livers was a transient phenomenon induced to counter vascular sinusoidal structure disruption during rapid hepatocyte regeneration triggered by DCE.

Caffeine and chlorogenic acid are the predominant polyphenol derivatives in coffee and their biological functions have been well investigated [33, 34], but many other constituents have not been characterized or clearly identified. Kahweol as a coffee-specific diterpene that has been reported to have anti-cancer properties. Kahweol-mediated cyclin D1 degradation may contribute to the inhibition of human colorectal cancer cell proliferation [35], and kahweol was observed to significantly decrease TGF-β (transforming growth factor beta) stimulated expressions of type I collagen and CTGF in vitro. In addition, in hepatocytes, kahweol significantly decreased the expressions of Smad3, STAT3, ERK, and JNK, which are involved in the induction of CTGF expression by TGF-β [36]. In the present study, DCE-2.5 reduced the protein levels of TGF-β1, TGF-β2, CTGF, SMAD4, STAT3, and ERK-1 by < ± 5%, but increased the expression of JNK-1 to 108.1%. Furthermore, this increase in JNK-1 co-occurred with increases in the levels of MMP-9, PI3K, and pAKT1/2/3, might suggest MMP-9 stimulated PI3K/Akt/JNK signaling was induced by DCE in mouse livers to support hepatocyte regeneration.

In the present study, DCE was found to increase levels of proliferation-related proteins, growth factors, cellular adaptation-related proteins, cellular differentiation-related proteins, inflammatory proteins, p53-mediated apoptosis-related proteins, angiogenesis-related proteins, and antioxidant and protection-related proteins; to reduce levels of NFkB signaling proteins, inflammatory proteins, and oncogenic proteins; but not to substantially effect levels of cMyc/MAX/MAD network proteins, p53/Rb/E2F signaling proteins, Wnt/β-catenin signaling proteins, epigenetic modification-related proteins, protein translation-related proteins, or FAS-mediated apoptosis-related proteins in mouse livers. Furthermore, global protein expression induced by DCE-5 was changed by < ± 10%, and thus, probably remained in the physiological homeostatic range. In addition, the levels of many essential proteins relevant to molecular signaling were increased or decreased by around ± 5% by DCE, which was similar to that observed for housekeeping proteins (β-actin, α-tubulin, and GAPDH). These changes suggest DCE-2.5-, DCE-5-, and DCE-10-treated mouse livers exhibited minimal cellular stress, inflammation, or chemical or oncogenic injury, and that after the rapid removal of senile or degenerated hepatocytes through p53-mediated apoptosis they underwent hepatocyte regeneration and sinusoidal vasculature recovery similar to wound healing mechanism.

## Conclusions

DCE upregulated the expressions of cellular proliferation, growth factor/RAS signaling, cellular protection, p53-mediated apoptosis, angiogenesis, and antioxidant-related proteins, but downregulated the expressions of NFkB signaling, inflammatory, and oncogenic proteins in mouse livers. These protein level changes usually fell in the range ± 10%, which suggested that murine hepatocytes reacted to DCE within thresholds of physiological homeostasis. DCE-2.5 and DCE-5 induced relatively mild dose-dependent changes in protein expressions as compared with non-treated controls, whereas DCE-10 induced fluctuations in protein expressions, which suggested DCE-2.5 and DCE-5 treatments were less stressful than DCE-10. Our IP-HPLC data also showed murine hepatocytes treated with DCE exhibited mild p53-mediated apoptosis, subsequent proliferation, and growth devoid of fibrosis signaling, which progressed to rapid cellular regeneration in the absence of inflammatory effects.

## Additional files

Additional file 1: A chromatography for LPS detection assay through IP-HPLC. Pink line: negative control using distilled water. Green line: experiment 1 to detect LPS in 1 mL DCE. Blue line: experiment 2 to detect LPS in 2 mL DCE. Red line: positive control using 1 mL LPS solution (1 μg/mL). The peak areas of experiments 1 and 2 were similar to that of negative control, while the peak area of positive control, LPS solution, was predominantly increased. These results may indicate that DCE is almost free from LPS contamination. (JPG 338 kb)

Additional file 2: Representative IP-HPLC chromatograms for the analysis of protein expression levels in the control and experimental groups. (JPG 739 kb)

Additional file 3: β-Actin expression in DCE-treated RAE 264.7 cells was explored through western blot and IP-HPLC. Densitometry data of triplicated western blot (red line) showed big standard deviation (16.1–23.2%), while triplicated IP-HPLC data (blue line) showed relatively small standard deviation (1.7–2.7%). Therefore, the latter was available to perform statistical analysis contrary to the former. (JPG 819 kb)

Additional file 4: Comparison of protein expression changes between western blot and IP-HPLC performed with same protein samples using IL–10, CD20, and NRAS antibodies. Western blot results showed relatively irregular protein expression changes depending on the increase of DCE dose, i.e., control, DCE-2.5, DCE-5, and DCE-10, compared to the IP_HPLC results. However, western blot data were plotted similar trends of protein expression to IP-HPLC data, but the protein expression changes of western blot data were not proportional and showed a feature of fluctuation in line graphs (A–C) compared to those of IP-HPLC data. (JPG 8955 kb)
