# Supplementary figures and images for: In vivo protein expression changes in mouse livers treated with dialyzed coffee extract as determined by IP-HPLC
Source: Maxillofac Plast Reconstr Surg. 2018 Dec 28;40(1):44. doi: 10.1186/s40902-018-0183-z (PMC6308107; doi:10.1186/s40902-018-0183-z)

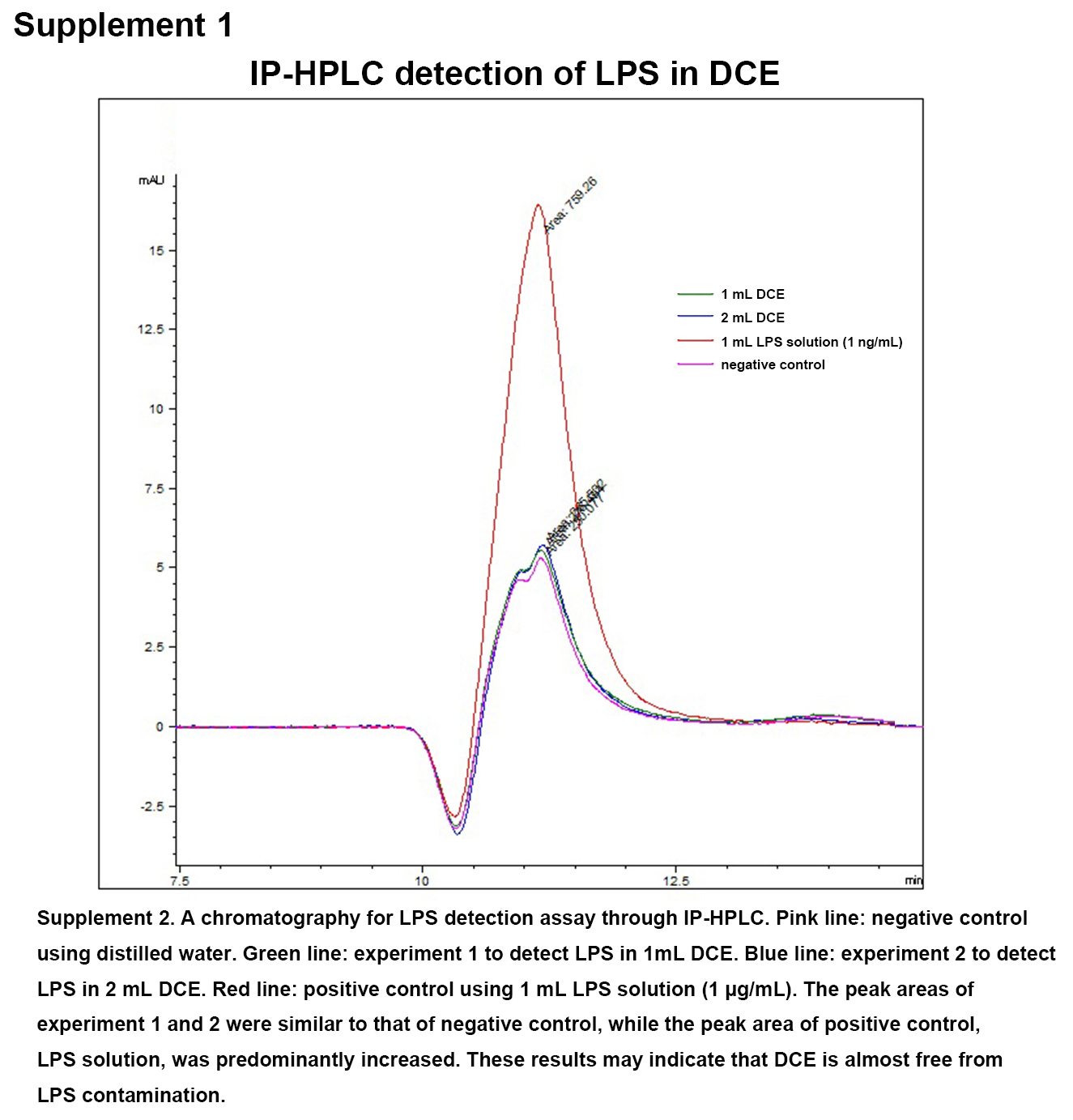

Supplement: Supplementary file 1 — A chromatography for LPS detection assay through IP-HPLC. Pink line: negative control using distilled water. Green line: experiment 1 to detect LPS in 1 mL DCE. Blue line: experiment 2 to detect LPS in 2 mL DCE. Red line: positive control using 1 mL LPS solution (1 μg/mL). The peak areas of experiments 1 and 2 were similar to that of negative control, while the peak area of positive control, LPS solution, was predominantly increased. These results may indicate that DCE is almost free from LPS contamination. (JPG 338 kb) [file 40902_2018_183_MOESM1_ESM.jpg]

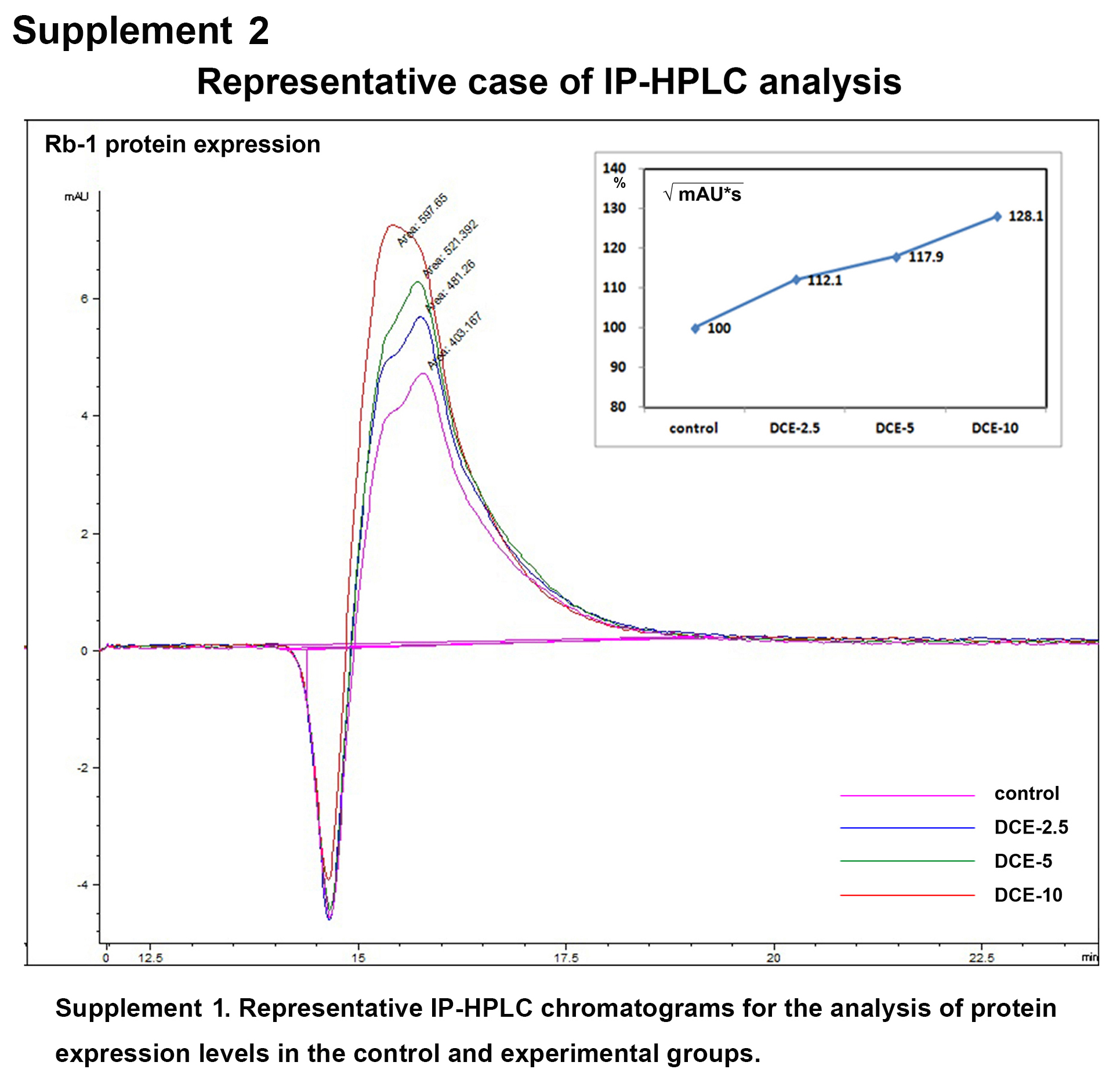

Supplement: Supplementary file 2 — Representative IP-HPLC chromatograms for the analysis of protein expression levels in the control and experimental groups. (JPG 739 kb) [file 40902_2018_183_MOESM2_ESM.jpg]

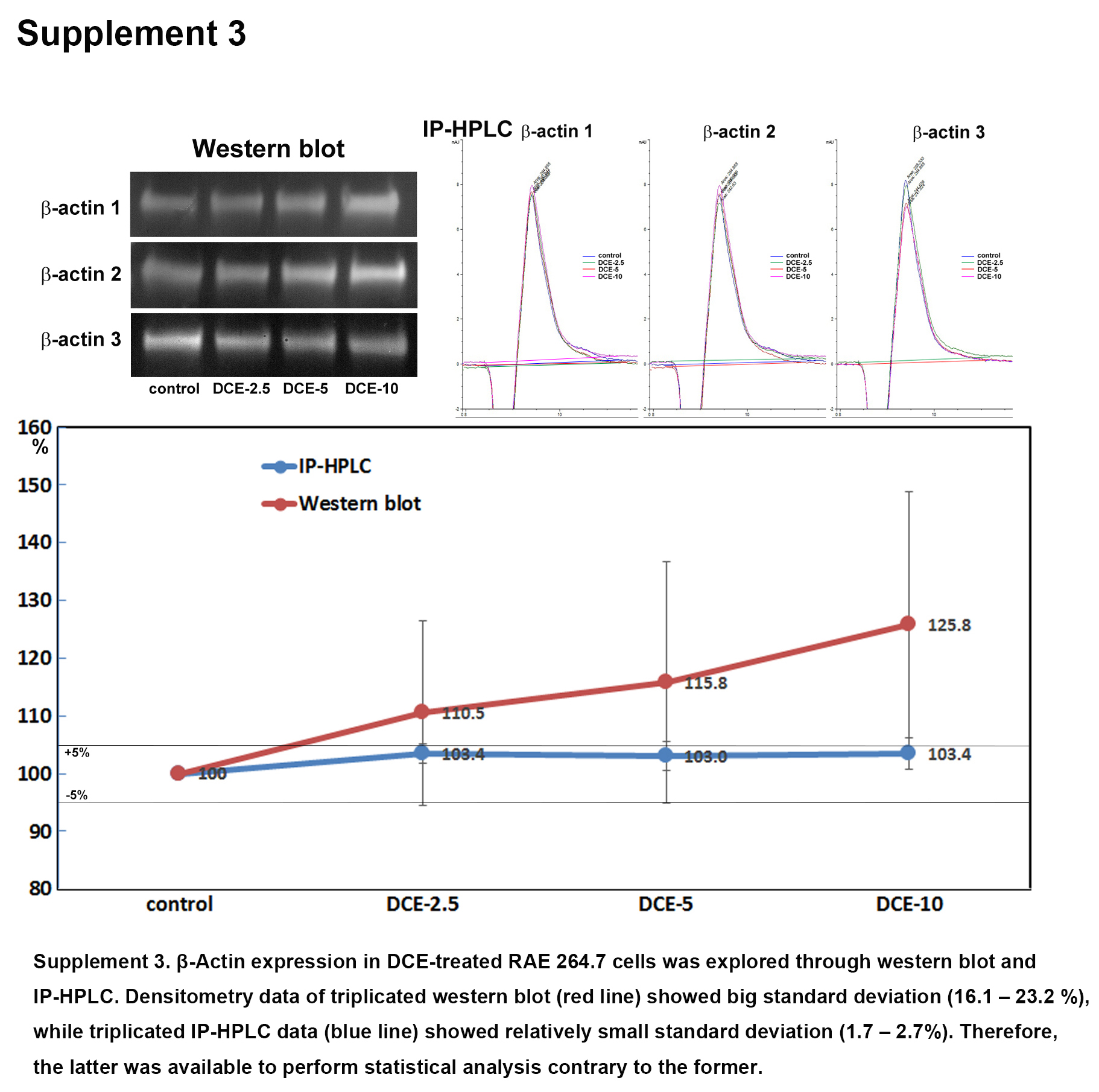

Supplement: Supplementary file 3 — β-Actin expression in DCE-treated RAE 264.7 cells was explored through western blot and IP-HPLC. Densitometry data of triplicated western blot (red line) showed big standard deviation (16.1–23.2%), while triplicated IP-HPLC data (blue line) showed relatively small standard deviation (1.7–2.7%). Therefore, the latter was available to perform statistical analysis contrary to the former. (JPG 819 kb) [file 40902_2018_183_MOESM3_ESM.jpg]

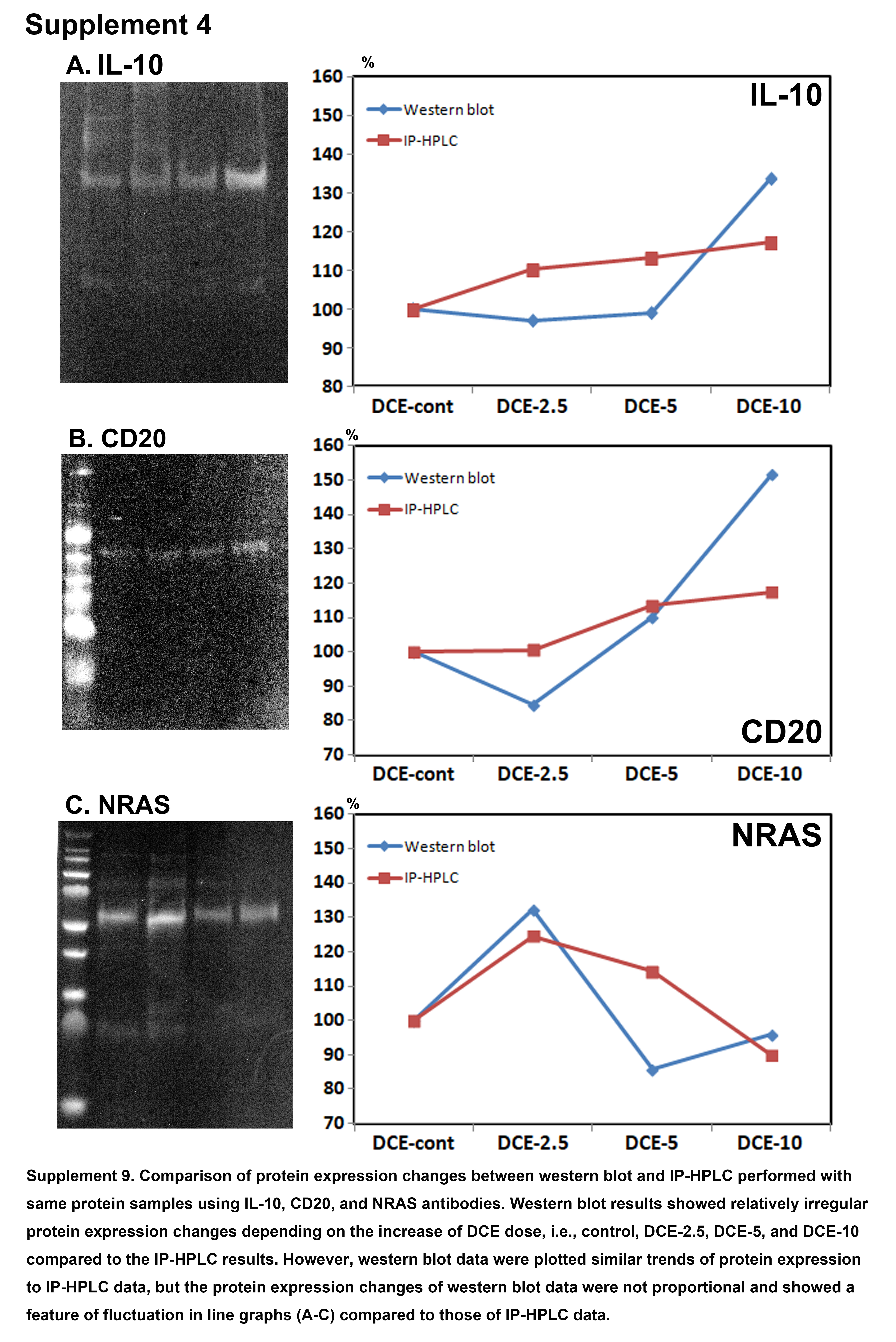

Supplement: Supplementary file 4 — Comparison of protein expression changes between western blot and IP-HPLC performed with same protein samples using IL–10, CD20, and NRAS antibodies. Western blot results showed relatively irregular protein expression changes depending on the increase of DCE dose, i.e., control, DCE-2.5, DCE-5, and DCE-10, compared to the IP_HPLC results. However, western blot data were plotted similar trends of protein expression to IP-HPLC data, but the protein expression changes of western blot data were not proportional and showed a feature of fluctuation in line graphs (A–C) compared to those of IP-HPLC data. (JPG 8955 kb) [file 40902_2018_183_MOESM4_ESM.jpg]
